# Students’ perceptions, engagement and satisfaction with the use of an e-rubric for the assessment of manual skills in physiotherapy

**DOI:** 10.1186/s12909-022-03651-w

**Published:** 2022-08-17

**Authors:** Silvia Pérez-Guillén, Andoni Carrasco-Uribarren, Carlos López-de Celis, Vanessa González-Rueda, Pere R. Rodríguez-Rubio, Sara Cabanillas-Barea

**Affiliations:** Department of Physical Therapy, Universitat Internacional de Catalunya, Barcelona, Spain

**Keywords:** Formative assessment, E-rubric, Physiotherapy

## Abstract

**Introduction:**

In recent years, formative assessment has gained importance in health care education to facilitate and enhance learning throughout the training period. Within the frame of active methodologies, rubrics have become an essential instrument for formative assessment. Most rubric-based assessment procedures focus on measuring the effects of rubrics on teachers. However, few studies focus their attention on the perception that students have of the evaluation process through rubrics.

**Methods:**

A cross-sectional survey study was carried out with 134 students enrolled in the pre-graduate Physiotherapy education. Assessment of manual skills during a practical examination was performed using an e-rubric tool. Peer-assessment, self-assessment and teacher´s assessment were registered. After completion of the examination process, students’ perceptions, satisfaction and engagement were collected.

**Results:**

Quantitative results related to students’ opinion about e-rubric based assessment, students’ engagement, perceived benefits and drawbacks of the e-rubric as well as the overall assessment of the learning experience were obtained. 86.6% of the students agreed upon the fact that “the rubric allowed one to know what it is expected from examination” and 83.6% of the students agreed upon the fact that “the rubric allowed one to verify the level of competence acquired”. A high rate of agreement (87.3%) was also reached among students concerning feedback.

**Conclusions:**

E-rubrics seemed to have the potential to promote learning by making criteria and expectations explicit, facilitating feedback, self-assessment and peer-assessment. The importance of students in their own learning process required their participation in the assessment task, a fact that was globally appreciated by the students. Learning experience was considered interesting, motivating, it promoted participation, cooperative work and peer-assessment. The use of e-rubrics increased engagement levels when attention was focused on their guidance and reflection role.

## Introduction

With the inclusion to the Common Space of Higher Education, an important structural, organizational and methodological adaptation of Spanish universities seemed necessary. One of the most relevant changes was the establishment of competencies [1]. In this way, the so-called competency training model arose, and the assessment process in higher education went through a shift from traditional testing of knowledge towards assessment for learning. The new assessment culture aimed at assessing higher-order thinking processes and competencies instead of factual knowledge and lower-level cognitive skills [2].

In the health care setting, competency-based medical education (CBME) has become a prevalently recommended approach to graduate and post-graduate medical education internationally during the last decades. This approach requires summative assessment (assessment of learning) at the end of the student´s training period to know the level of competence and assure that competencies have been achieved to a certain standard [3].

However, in recent years, formative assessment has gained importance in health care education to facilitate and enhance learning throughout the training period [4]. Formative assessment (assessment for learning) aims to identify students’ strengths and weaknesses and to be conducive to progress by means of identifying learning needs and providing feedback in the sense of giving information about the difference between a students’ current level of skills and a given standard [5]. Although assessment always has a summative aspect, formative assessment has the potential to provide feedback and give direction for further development.

The use of rubrics as part of active learning pedagogies.

Within the frame of active learning pedagogies, rubrics have become an essential instrument for formative assessment. Rubrics appear to have the potential to promote learning because they make expectations and criteria explicit, facilitating feedback, self-evaluation and peer-assessment [1].

A rubric can be defined as the set of quality criteria related to the competence or competencies to be evaluated, determined by descriptors that imply different levels of achievement or performance. Rubrics can be easily understood and applied by teachers and students, even external evaluators [6, 7]. Rubrics are a very useful tool to encourage student´s feedback as well as to improve their expectations regarding the evaluation process. They allow students to check if their results coincide with the teachers' expectations, in order to avoid potential disappointment or frustration if the result of the work carried out is not exactly as it was expected [8].

Thanks to recent developments in the field of technology, students have become more involved, and aware of their own assessment process and very interesting assessment modalities have emerged from a cooperative point of view, such as peer evaluation and self-assessment. All of them have enabled students to be better judges of their own work. As our students are increasingly working in technology-based environments, the shift from a normal rubric to an e-rubric gives the following advantages: a) it is easier to use; b) feedback, which is one of the central points of formative assessment, can be given much more quickly; c) students can better self- regulate their learning process; d) they provide more interaction and; e) they foster students’ autonomy in the evaluation of their competences [9, 10].

In a peer-assessment activity, students take responsibility for assessing the work of their peers against set assessment criteria. In formative peer assessment, the intention is to help students help each other when planning their learning. The students expand their knowledge in a social context of interaction and collaboration according to social constructivism principles [11–13]. Self-assessment has been introduced in the classroom with the intention of promoting students’ monitoring and self-evaluation strategies as it usually leads to a deeper understanding of the academic tasks [14].

### The use of rubrics in physiotherapy education

Clinical education is defined as the provision of guidance and feedback on personal, professional and educational development in the student's experience of providing appropriate patient care [15]. Appropriate clinical education within the context of providing patient care is important for the development of health professionals [16–18].

In physical therapy (PT), as in other health-related disciplines, professionals have to master competencies from different specialties. One of these is manual therapy (MT), and it could be considered one of the most complex specialty, not only because it constitutes in itself a recognized area of ​​specialization within PT but because the acquired competencies are transversal to other areas of knowledge (pediatric physiotherapy, neurological physiotherapy, sports physiotherapy, …) [19, 20].

Many studies highlight the importance of developing manual skills in a broad set of PT subjects because they are essential in the professional world [17, 21]. This is the case of MT, where students have to acquire and fluidly apply a wide range of different techniques and maneuvers both on colleagues and patients [22, 23] in order to be effective when assessing and treating patient´s pain experience [24]. In addition, the process of learning MT techniques and maneuvers is more demanding than in other PT areas, given its greater breadth, diversity, and specificity. Consequently, it is particularly relevant to provide students with different types of support to promote their learning, especially when we deal with assessment processes. In this regard, instructional or formative rubrics can be particularly useful resources because they provide students with the criteria and performance levels to be reached. This is the reason why they are widely used in the academic setting, not only within PT but in other healthcare degrees (nursing, psychology,..) [25].

Despite its importance, there is limited in-depth knowledge about which assessment strategies are potentially effective to facilitate learning and why some strategies might be more effective than others [26]. Ernstzen et al*.,* [27] found that certain learning opportunities such as demonstrations and discussion on patient management are suitable to provide feedback on clinical skills. However, few studies focus their attention on students' perception of the evaluation process through rubrics.

Most rubric-based assessment procedures focus on measuring the effects of rubrics on teachers (since they are in charge of their production) and how they improve the teaching process. These studies usually conclude that the teachers' perceptions are positive in terms of increasing the transparency of the evaluation process and that they constitute a facilitating element of the evaluation process [28, 29].

However, few studies focus their attention on the perception that students have of the evaluation process through rubrics. And this constitutes a fundamental element in the correct development of the evaluation process, since the perceptions and attitudes that students show towards this evaluation instrument are key to the success of the evaluation [30, 31].

It is hypothesized that knowing students' opinions about the assessment process with the e-rubric would allow us to understand the potential that this type of assessment process has on students' learning experience. Thus, the objectives of the current study are: 1) to describe and understand students’ opinion about the experience in the use of e-rubrics for the evaluation of practical skills in the context of MT; 2) to describe and understand students’ satisfaction with the e-rubrics based evaluation process; 3) to describe and understand students’ engagement with the e-rubrics based evaluation process and; 4) to describe the advantages and disadvantages of the e-rubrics based evaluation process as experienced by students.

## Materials and methods

### Study design and sample

A cross-sectional survey study was carried out. The Ethics Committee of Universitat Internacional de Catalunya (UIC) approved the research protocol for the study (Code FIS-2021–10).

The study inclusion criteria were to be a student enrolled in Manual Therapy I and II subjects, presenting to the first practical examination (May 2021). These subjects were included as part of the programme of the second and third year of the Physiotherapy Degree at UIC in the 2020–2021 academic year. The classes last one semester, and their practical part consists of 20 h of face-to-face instruction that take place in laboratories in groups of 18–20 students. This practical part ends with a practical examination which represents the context in which this study is developed.

#### Study procedure

Practical examinations took place on the 6^th^ and 7^th^ of May 2021. Before the exam, students were informed about the examination, procedure and the rubric used for evaluating practical performance was available for them. This rubric was designed by the six teachers involved in the Manual Therapy subjects and consisted of five items (“patient's position”, “PT´s position”, “execution procedure”, “effect” and “clinical reasoning”) which were assessed according to four levels of skill´s performance (“expert”, “proficient”, “competent” and “novel”). Description of items and levels is included in Table 1. The rubric was delivered to students in an e-rubric format using CoRubrics, an add-on for Google Sheets that facilitates the assessment process [32].

**Table 1:** Items and levels of the e-rubric

	**EXPERT**	**PROFICIENT**	**COMPETENT**	**NOVEL**
**PATIENT'S POSITION**	Nothing to improve (patient is in good ergonomics and feels comfortable)	Student forgets small details	Patient does not feel comfortable or the patient's position is not optimal for the selected technique	Student does not know how to position the patient
**PT´S POSITION**	Nothing to improve (good ergonomics and good use of body)	Body movement can be improved, but ergonomics and positioning regarding patient are good	Student knows where and how to be placed but ergonomics is not good	Student does not know where and how to be placed and ergonomics is poor
**PROCEDURE**	Nothing to improve	Technique is performed correctly but some aspects should be improved (direction, intensity, grips,…)	Student knows the selected technique but does not perform it correctly (there is poor correlation between what the student says and performs)	Student does not know the selected technique
**EFFECT**	Desired effect for the selected technique	Technique is effective but effect can be improved by modifying some parameters (direction, intensity, grips,…)	Effect does not reach the targeted structure/segment/region	No effect or contraindicated effect
**CLINICAL REASONING**	Student knows the clinical presentation, indication of the technique, technique´s adequation to clinical presentation and how to solve problems and incidents	Student fails in one of the following items: indication of the technique, technique´s adequation to clinical presentation and how to solve problems and incidents	Student knows the technique but is not able to solve problems	Student is not able to describe the technique or its use and indications

On the examination day, students were divided into groups of 8–10 members. One teacher supervised each group. One by one, each student within the group shall perform a manual therapy technique on a colleague. In the meantime, the rest of the students in the group shall evaluate their performance (co-evaluation) according to the criteria in the rubric previously mentioned. The same was done by the teacher (teacher-evaluation) and the student being evaluated (self-evaluation).

Once all students in the group finished their examination, they were invited to respond to an anonymous online survey about their perceptions of the e-rubric based evaluation process, as well as their level of engagement and satisfaction. Questions related to sociodemographic and educational variables were also included. The first page of the survey described the study characteristics and objectives and requested students´ informed consent to complete the survey.

#### Measures

The standardized questionnaire “Students´ opinion about the e-rubric based evaluation process” developed by Rasposo and Martinez [33] was administered to students in the Spanish language.

This questionnaire consists of two sections:

- Sect. 1 includes 11 “close-answer” items to evaluate the degree of agreement-disagreement of the students using a Likert scale of four answer options. Its reliability is 0.814 (Cronbach's Alpha). This first section covers the following dimensions: rubric characteristics, assessment modality, assessment procedure and learning impact.
- Sect. 2 consists of 9 items, with a 0–10 rating scale. Its reliability is 0.716 (Cronbach's Alpha). It covers the following dimensions: student engagement and students´ global perception of the assessment process.

Finally, a questionnaire developed ad hoc for the study was administered to collect the participants’ sociodemographic and educational variables (course of the degree in which they were enrolled, university entrance modality and subjects pending from previous years).

#### Analysis

For statistical analysis, IBM SPSS Statistic 25.0 software was used. Descriptive analysis was carried out. For quantitative variables, mean and standard deviation were calculated. Frequencies were calculated for qualitative variables.

## Results

Of the 164 students enrolled in the course, 134 presented to the first practical examination (69 students were enrolled in the subject Manual Therapy 1 and 65 students in Manual Therapy 2). All of them agreed to participate in the study. Their mean age was 22.29 ± 2.84 years, with a similar distribution of sex (46.3% men and 53.7% women). Of the total number of participants, 93.3% had entered university studies through the baccalaureate degree and EBAU tests and only 8.2% of the students had pending subjects from previous courses.

The analysis of the information collected with the described instrument has made it possible to obtain quantitative results related to the following aspects: students’ opinion about e-rubric based assessment, students’ engagement, perceived benefits and drawbacks of the e-rubric as well as the overall assessment of the learning experience.

### Students’ opinion on the e-rubric-based assessment

Satisfaction/dissatisfaction both with the different functions of the rubric and the e-rubric assessment process were measured with a four-grade rating scale: Totally Disagree (TD), Disagree (D), Agree (A) and Strongly Agree (SA). Results were organized around four dimensions:

1. Rubric characteristics:

The ability of the rubric to verify students’ level of performance was registered under two statements as shown in Table 2. 86.6% of the students agreed or fully agreed upon the fact that “the rubric allowed one to know what it is expected from examination”, versus 13.4% of the students who disagreed or fully disagreed. Regarding the second statement, 83.6% of the students agreed or fully agreed upon the fact that “the rubric allowed one to verify the level of competence acquired”, versus 16.4% of the students who disagreed or fully disagreed (Table 2).

- (2) Assessment modality:

**Table 2:** Rubric characteristics

	Fully disagree	Disagree	Agree	Fully agree
A. The rubric allowed one to know what it is expected from examination	0.7%	12.7%	65.7%	20.9%
B. The rubric allowed one to verify the level of competence acquired	4.5%	11.9%	61.2%	22.4%

During practical examinations students were assessed both by themselves, and their colleagues. These results are shown in Table 3. Regarding the ability of the rubric to perform self-assessment, 86.5% of the students agreed or fully agreed, and just 13.4% of them disagreed or fully disagreed. Concerning the adequacy of the rubric for peer-assessment (co-evaluation), 94.0% of the students agreed or fully agreed, and just 5.9% of them disagreed or fully disagreed. The third item gathered regarding assessment modality was equality in assessing every group/student. 70.9% of the students agreed or fully agreed with this item, and 29.1% of them disagreed or fully disagreed (Table 3).

- (3) Assessment process:

**Table 3:** Assessment modality

	Fully disagree	Disagree	Agree	Fully agree
A. The rubric allowed self-assessment	0.7%	12.7%	52.2%	34.3%
B. The rubric allowed peer-assessment	0.7%	5.2%	60.4%	33.6%
C. The rubric allowed to assess every group/student equally	5.2%	23.9%	47.8%	23.1%

The main issue included in this section of the assessment process is transparency, which was collected under the following statements. As shown in Table 4, first, the objectiveness of evaluation was assessed, and 76.1% of the students agreed or fully agreed upon the fact that the rubric allowed a more objective assessment, versus 23.9% of the students who disagreed or fully disagreed. A second statement gathered students’ opinions about whether the rubric made teachers clarify their examination objectives, and a rate of agreement and full agreement of 84.4% was reached among students. Just 15.7% of the students disagreed or fully disagreed. The third statement asked students whether the rubric showed how they would be assessed, and 94.6% of the students agreed or fully agreed. Finally, 74.6% of the students agreed or fully agreed upon the fact that the rubric demonstrated the work done, versus 25.4% of the students who disagreed (Table 4).

- (4) Learning impact

**Table 4:** Assessment process

	Fully disagree	Disagree	Agree	Fully agree
A. The rubric allows a more objective assessment	4.5%	19.4%	58.2%	17.9%
B. The rubric makes teachers clarify the criteria	3.0%	12.7%	57.5%	26.9%
C. The rubric shows how we will be assessed	0%	5.2%	63.3%	31.3%
D. The rubric demonstrates the work done	3.0%	22.4%	61.9%	12.7%

The ability of the rubric to provide feedback and help students understand the features of the examination were the two issues assessed concerning the learning impact. Table 5 shows a high rate of agreement (87.3%) reached among students concerning feedback. However, still 12.7% of the students did not agree or fully disagree with the fact that the rubric provides feedback. Finally, 91.1% of the students agreed or fully agreed upon the fact that the rubric helped them understand the features of the assessment process, and 8.9% disagreed or fully disagreed (Table 5).

**Table 5:** Learning impact

	Fully disagree	Disagree	Agree	Fully agree
A. The rubric provides feedback	1.5%	11.2%	61.2%	26.1%
B. The rubric helps us understand the features the examination shall have	2.2%	6.7%	67.2%	23.9%

### General assessment of experimentation with rubrics

The analysis of the students' responses regarding the evaluation of the whole learning experience is reflected in Tables 6 and 7. Both direct scores (on a double interval scale from 1 to 10) and mean values are presented. Items have been organized in two main dimensions: students’ engagement and students’ perceptions about the assessment process.

- (1) Students’ engagement

**Table 6:** Students’ engagement

	1–2 points	3–4 points	5–6 points	7–8 points	9–10 points
A. The rubric has motivated me	9.0%	9.7%	20.1%	37.3%	23.9%
B. The rubric has promoted participation	6.7%	9.7%	19.4%	36.6%	27.6%
C. The rubric has made me more responsible	11.9%	8.2%	25.4%	42.5%	11.9%
D. I have performed collaborative work within the group	6.7%	9.0%	17.2%	35.8%	31.3%
E. I have cheated	74.6%	6.0%	12.7%	6.0%	0.7%

**Table 7:** Students’ global perceptions about the assessment process

	1–2 points	3–4 points	5–6 points	7–8 points	9–10 points
A. Peer-assessment with rubric “Has been very interesting”	9.7%	11.2%	15.7%	35.8%	27.6%
B. Peer-assessment with rubric “Has been very good”	10.4%	11.2%	16.4%	34.3%	27.6%
C. Peer-assessment with rubric “Is not useful”	59.7%	13.4%	18.7%	5.2%	3.0%

The aspect achieving the greater score refers to: “I have performed collaborative work within the group” (with 84.3% of the students scoring above 5 points on the 0 to 10 scale and a mean value 7.48 points; SD 2.36). It is closely followed by the aspects “the rubric has promoted participation” (with 83.6% of the students scoring above 5 points on the 0 to 10 scale and a mean value 7.31 points; SD 2.35), “the rubric has motivated me” (with 81.3% of the students scoring above 5 points on the 0 to 10 scale and a mean value 6.83 points; SD 2.51) and “the rubric has made me more responsible” (with 79.8% of the students scoring above 5 points on the 0 to 10 scale and a mean value 6.68 points; SD 2.35). It should be noted that a very high percentage (80.6%) recognizes not cheating (1–2 points on the 1 to 10 scale) or doing little cheating (3–4 points on the 1 to 10 scale) during the assessment process (Table 6).

- (2) Students´ global perceptions about the assessment process:

The most highlighted aspect by the students about the assessment process is peer-assessment (co-evaluation), which they found very good (78.3% of the students scoring above 5 points on the 1 to 10 scale) and interesting (79.1% of the students scoring above 5 points on the 1 to 10 scale), with mean values of 7.15 (SD 2.58) and 7.21 (SD 2.54) points, respectively.

The results show that 73.1% of the students did not agree on the fact that peer assessment with the e-rubric was not useful, scoring below 5 points in the 1 to 10 scale (mean value of 2.57 points; SD 2.21) (Table 7).

## Discussion

Our students agreed that the rubric allowed them to know what was expected from the examination and also agreed on the fact that the rubric allowed them to verify the level of competence acquired. Judging from the data, rubrics appear to have the potential to promote learning because they make explicit expectations and criteria, facilitating feedback and self-assessment [1]. This is in line with the results obtained in the study carried out by Reynolds et al*.* [34] in which students claimed that they better understood teacher expectations when the assignment involved a rubric. As stated by Panadero et al*.* [35]*,* students’ anxiety (negative self-regulated learning) may decrease when implementing long-term interventions with rubrics, which is probably due to the fact that students know what is expected of their work and how it will relate to their grades.

Great agreement was observed among our students regarding the usefulness of the e-rubric for self-assessment. This is an important fact as one of the implicit goals of higher education is to enable students to be better judges of their own work [9]. As Martínez-Figueira et al*.,* [36] pointed out, the use of rubrics for self-assessment raises levels of engagement and probably learning levels. Self- assessment also facilitates students’ understanding of the learning process, contrasting their achievements against objective proof presented by the e-rubrics.

Similar results were obtained regarding peer-assessment, as a high percentage of the students involved in our study stated that the e-rubrics allowed assessment between colleagues. Peer assessment counts on a wide literary tradition that is enhanced by the use of e-rubrics. This type of assessment facilitates peer correction, information feedback and peer analysis of the processes involved [37–39].

About 60% of the students agreed upon two facts: that it makes teachers clarify the criteria and shows how they will be assessed. However, nearly 20% of the students did not agree that the rubric allows for a more objective assessment, and that it demonstrates the work done. This can be due to the fact that the application of assessment criteria differs according to whether it is interpreted by teachers or students [40]*.* Maybe working together with students on criteria formation and adoption will make students active in the process and increase the success rate of the peer assessment [41, 42].

Regarding learning impact, again over, 60% of the students agreed that the rubric provided feedback. This is in line with other studies which indicate that e-rubrics contribute to student learning by aiding the feedback process [43]. And give more informative feedback about their strengths and areas in need of improvement [44]. Some authors argue that this positive effect on learning may be affected by the motivation and satisfaction students show with the use of technology in general [45].

Engagement was one of the main outcomes of our study. Students agreed that the rubric had motivated them, also made them participate more and increased their responsibility on their own learning process. This is in line with the results obtained by Hanrahan et al*.,* [46] which showed that throughout the peer assessment process, students learn to develop high levels of responsibility and to focus on learning itself. And that peer assessment also provides the learners with a context where they can observe the role of their teachers and understand the role of assessment [47].

Results showed that the e-rubric based evaluation process has allowed students to perform collaborative work. Although nearly 75% of the students did not cheat during the assessment process, the remaining 20% did. This may be due to the fact that students often have a negative attitude towards peer assessment. Some students may not like the idea of having their work to be assessed by peers or assessing their peers’ work as they may feel less capable than their colleagues in achieving a certain standard or may think that increasing their colleagues’ grade may make their mates also increase theirs. This could have made students perform some more cheating [48].

Finally, regarding the global perception of the assessment process, students showed high interest and satisfaction with the rubric. These results could have been achieved due to the fact of working with technology and that teachers explained the purpose of the assessment and that a couple of sessions to get familiar with the rubric were performed. This could have helped students become more confident about themselves and their peer assessors [48].

One of the limitations of the study could have been that the rubric was used in only one examination. The literature shows that in studies where the rubric was introduced during one period only, or where the students got only a couple of lessons in self-assessment, the effects reported are small and only partial. Other limitations of the study are those derived from the context in which the examination was developed. The rubric was designed specifically for addressing manual skills within the subjects of Manual Therapy and students from two different courses included in the study data were not analyzed separately. A variation in the results could have been seen as students enrolled in the subject Manual Therapy II are one course above those in Manual Therapy I and may be more familiar with the use of these type of assessment tools as they are also used in other subjects.

## Conclusions

E-rubrics seem to have the potential to promote learning by making criteria and expectations explicit, facilitating feedback, self-assessment and peer-assessment. The importance of students in their own learning process requires their participation in the assessment task, a fact that is globally appreciated by the students. Information analysis gathered by the instrument described has allowed us to confirm that the learning experience has been considered interesting, motivating, it has promoted participation, cooperative work and peer-assessment. Transparency and clarity items seem to concern students, issues which are not solved by the use of an instrument. The use of e-rubrics increases engagement levels when attention is focused on their guidance and reflection role.

## Data Availability

The data sets generated and analyzed during the current study are not publicly available because the local ethics board requires that members of the research team keep them securely. Could be shared the dataset upon reasonable request to the corresponding author.

## Abbreviations

CBME: Competency-based medical education
UIC: Universitat Internacional de Catalunya
TD: Totally Disagree
D: Disagree
A: Agree
SA: Strongly Agree
